# Structure-Mediated Excitation of Air Plasma and Silicon Plasma Expansion in Femtosecond Laser Pulses Ablation

**DOI:** 10.1155/2018/5709748

**Published:** 2018-12-09

**Authors:** Qingsong Wang, Lan Jiang, Jingya Sun, Changji Pan, Weina Han, Guoyan Wang, Feifei Wang, Kaihu Zhang, Ming Li, Yongfeng Lu

**Affiliations:** ^1^Laser Micro/Nano Fabrication Laboratory, School of Mechanical Engineering, Beijing Institute of Technology, Beijing 100081, China; ^2^Beijing Engineering Research Center of Applied Laser Technology, Institute of Laser Engineering, Beijing University of Technology, Beijing 100124, China; ^3^Beijing Spacecrafts, China Academy of Space Technology, Beijing 100094, China; ^4^State Key Laboratory of Transient Optics and Photonics, Xi'an Institute of Optics and Precision Mechanics, Chinese Academy of Sciences, Xi'an 710119, China; ^5^Department of Electrical and Computer Engineering, University of Nebraska-Lincoln, Lincoln, NE 68588-0511, USA

## Abstract

Femtosecond laser-induced surface structures upon multiple pulses irradiation are strongly correlated with the pulse number, which in turn significantly affects successive laser-material interactions. By recording the dynamics of femtosecond laser ablation of silicon using time-resolved shadowgraphy, here we present direct visualization of the excitation of air plasma induced by the reflected laser during the second pulse irradiation. The interaction of the air plasma and silicon plasma is found to enhance the shockwave expansion induced by silicon ablation in the longitudinal direction, showing anisotropic expansion dynamics in different directions. We further demonstrate the vanishing of air plasma as the pulse number increases because of the generation of a rough surface without light focusing ability. In the scenario, the interaction of air plasma and silicon plasma disappears; the expansion of the silicon plasma and shockwave restores its original characteristic that is dominated by the laser-material coupling. The results show that the excitation of air plasma and the laser-material coupling involved in laser-induced plasma and shockwave expansion are structure mediated and dependent on the pulse number, which is of fundamental importance for deep insight into the nature of laser-material interactions during multiple pulses ablation.

## 1. Introduction

Femtosecond laser-induced plasma, as a critical process of complicated laser-material interactions, has attracted intense attention concerning the fundamental mechanisms of laser ablation and the practical applications in micro/nanofabrication [1–3], nanoparticle synthesis [4, 5], thin film deposition [6, 7], and laser-induced breakdown spectroscopy [8, 9]. One remarkable characteristic of femtosecond laser-induced plasma is the absence of laser-plasma interactions because of the ultrashort pulse duration [10], making the behavior of the plasma differ significantly from that induced by nanosecond laser ablation. To acquire the plasma dynamics, several diagnostic techniques have been developed [11–14], among which time-resolved shadowgraphy with femtosecond temporal resolution and micrometer spatial resolution has been widely employed. In particular, shadowgraphy images of transient plasma structure and material ejection provide insights into thermal and nonthermal laser ablation mechanisms [15–19]. Further analysis of the time-dependent expansion of plasma and subsequent shockwave based on the point explosion theory provides an estimation of the energy conversion, as demonstrated in silicon ablation [20, 21]. In addition, shadowgraphy image also gives insight into the plasma dynamics generated under different ablation conditions, such as the variation of thickness of thermally grown oxide films [22, 23], air pressures [24], and in the case of excitation of air plasma at high laser intensity [24–26]. However, the aforementioned understanding of the plasma dynamics was mainly conducted with single-pulse irradiation, and the plasma dynamics during multiple pulses laser ablation was habitually ignored and still remains unclear. Recent studies have indicated that the structures generated by prior pulses can interact with subsequent laser and reshape the intensity distribution, thereby affecting the ablation process [27–29]. Our previous study demonstrated the enhancement of plasma and shockwave expansion during femtosecond two-pulse ablation [30]. This phenomenon was theoretically interpreted with laser-induced air breakdown enhanced by crater-induced laser refocusing but was limited by the lack of direct experimental observation of the excitation of air filaments. In addition, recent studies have suggested that the morphology of the laser-induced crater becomes rough as the pulse number (*N*) increases [31–34], leading to the loss of laser refocusing ability and can significantly alter the nature of the expansion of the laser-induced plasma and shockwave.

Herein, we investigate how laser-induced plasma and shockwave from silicon ablation vary as a function of incident pulse number using time-resolved shadowgraphy. We present a direct experimental visualization of the excitation of air plasma on femtosecond timescale induced by the reflected pulse, which is mediated by the crater structure generated by the previous pulse. The expansion of laser-induced plasma and shockwave in the scenario is systematically analyzed. The results reveal two fundamental mechanisms that determine the expansion of plasma and shockwave: the excitation of air plasma with an anisotropic expansion effect and laser-material coupling with an isotropic expansion effect. The dominant mechanism is determined by the morphology of laser-induced crater, which is strongly dependent on the pulse number.

## 2. Results

### 2.1. Two Pulses Ablation

**Figure 1:**
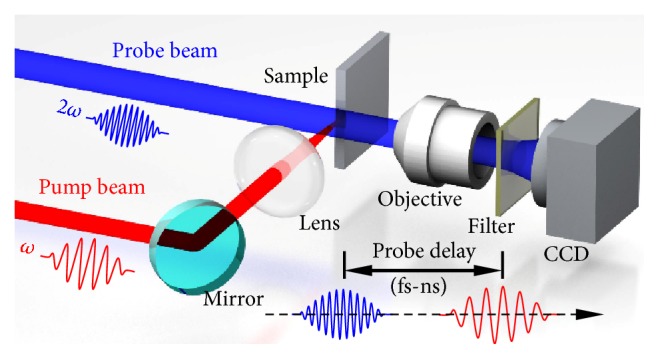
Schematic of the pump-probe setup.

**Figure 2:**
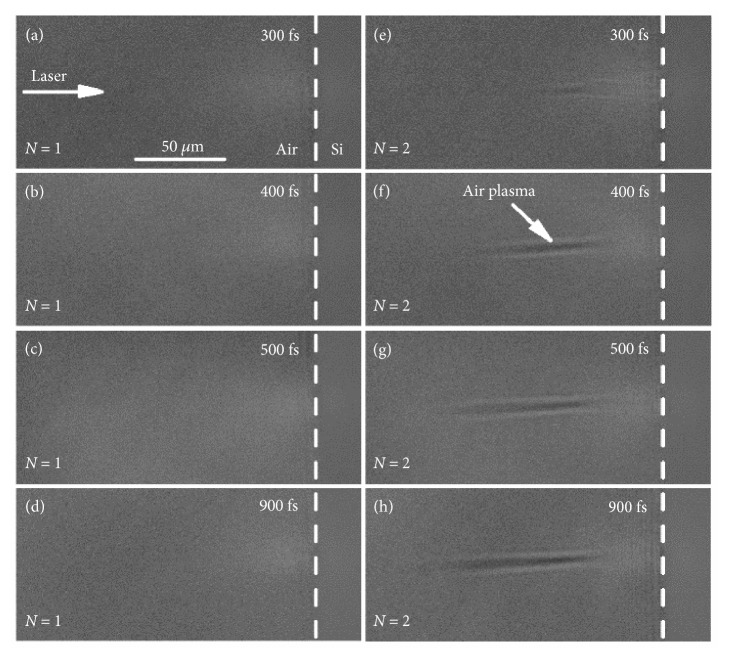
Time-resolved shadowgraphs of the laser ablation of silicon at probe delays on femtosecond timescale. (a–d) Shadowgraphs for the first pulse. (e–h) Shadowgraphs for the second pulse. The laser fluence is 7.27 J/cm^2^. The dashed line indicates the interface of silicon and air. The white arrow in (a) indicates the propagation direction of the incident laser.

We first focus on the plasma and shockwave evolution recorded during the first two pulses ablation at probe delays ranging from femtoseconds to nanoseconds with ultrafast pump-probe shadowgraphy (Figure 1). The pump pulse separation exceeded one second and the laser fluence was set at a constant of 7.27 J/cm^2^.  Figure 2 shows the transient shadowgraphs of the first two pulses ablation of silicon at probe delays on femtosecond timescale. No transient phenomena were observed to emerge and the shadowgraph remained unchanged on femtosecond timescale during the first pulse irradiation (Figures 2(a)–2(d)). However, a pronounced narrow dark region was clearly visible at a probe delay of 300 fs during the second pulse irradiation (Figure 2(e)). The length of the dark region extended with direction opposite to the incident direction of the pump pulse with the increase of probe delay (Figure 2(f)). For larger probe delays, the length of the dark region reached saturation; meanwhile, the overall shape and the transmission of the dark region remained nearly unchanged (Figures 2(g)–2(h)). The propagation velocity of the dark region recorded at probe delays from 300 fs to 400 fs was estimated to be 2.91 × 10^8^ m/s, which is close to the velocity of light in air. The distinctive features of the dark region indicate the excitation of air plasma induced by the reflected beam during the second pulse irradiation. The laser-induced air plasma can absorb the probe beam, thus resulting in the dark region in the shadowgraphs.

**Figure 3:**
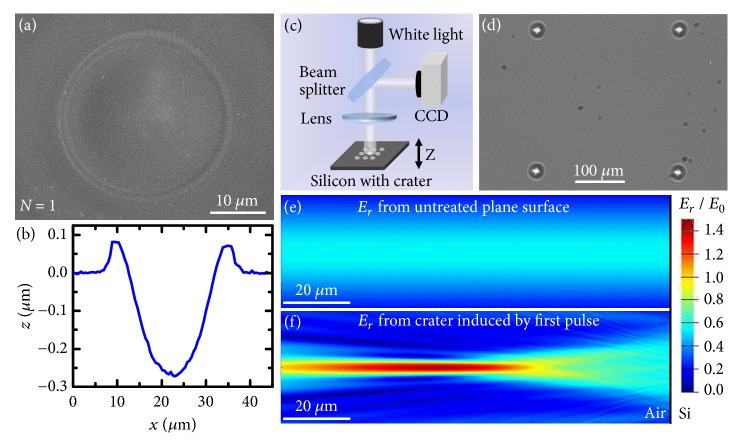
Characterization of the ablation morphology induced by the first pulse with a fluence of 7.27 J/cm^2^. (a) SEM image of the surface structure of silicon. (b) Atomic force microscope (AFM) profile of the cross-section of the structure in (a). (c) Sketch of the optical system for characterizing light focusing ability of the silicon-based crater array. (d) Focal spots image obtained with a CCD on the focal plane of the crater. (e) Calculated electric field distribution of the reflected pulse (*N* = 1) from untreated plane silicon surface. (f) Calculated electric field distribution of the reflected pulse (*N* = 2) from the crater generated by the first pulse. The focal spot of the incident pulse in (e) and (f) was set to be on the sample surface, indicated by right edge of the images.

In order to reveal the formation mechanism of air plasma, we have analyzed the ablation morphology induced by the first pulse. The scanning electron microscope (SEM) image of the laser-induced structure formed on silicon upon single-pulse irradiation is shown in Figure 3(a). A smooth and symmetric crater-shaped structure was generated on the silicon surface with a rim formed around the crater. The diameter and depth of the laser-induced crater were approximately 19.4 *μ*m and 0.27 *μ*m, respectively (Figure 3(b)). Details of the crater suggest that it can be considered as a concave mirror, which will reflect and focus the next incident pulse. Figure 3(d) shows the optical reflection image of the silicon-based crater array captured with an optical microscopy system (Figure 3(c)); the crater was illuminated with a white light. On the focal plane of the crater, an array of bright focal spots was visible in the crater center, which indicates excellent light focusing ability. Furthermore, the electric field distribution of the second pulse reflected by the preformed crater was simulated with the finite difference time domain method; the focal spot of the incident pulse was set to be on the sample surface. For simplicity, we only conducted a two-dimensional simulation with the complex refractive index of silicon at 800 nm held constant at* n* = 3.692 +* i*0.0065 [35]. Figure 3(f) shows that a refocusing region was generated above the silicon surface due to the focus effect of the crater as compared with the calculation result for the untreated plane silicon surface (Figure 3(e)). Moreover, the calculated electric field intensity of the refocused pulse can be stronger than the incident field intensity. In fact, free electrons can be generated on the silicon surface via linear and nonlinear ionization by the rising edge of the pulse during intense femtosecond laser irradiation, which results in a huge increase in transient reflectivity to significantly reflect the latter part of the pulse according to the Drude model [36]. Thus, the real field intensity of the refocused laser is much larger than the calculated intensity. The calculated electric field distribution is consistent with the morphology of the air plasma observed in our experiment. The analyses demonstrate the excellent light focusing ability of the crater induced by the first pulse, which is able to focus the reflected pulse and generate a stronger laser intensity to excite air plasma during the second pulse irradiation.

**Figure 4:**
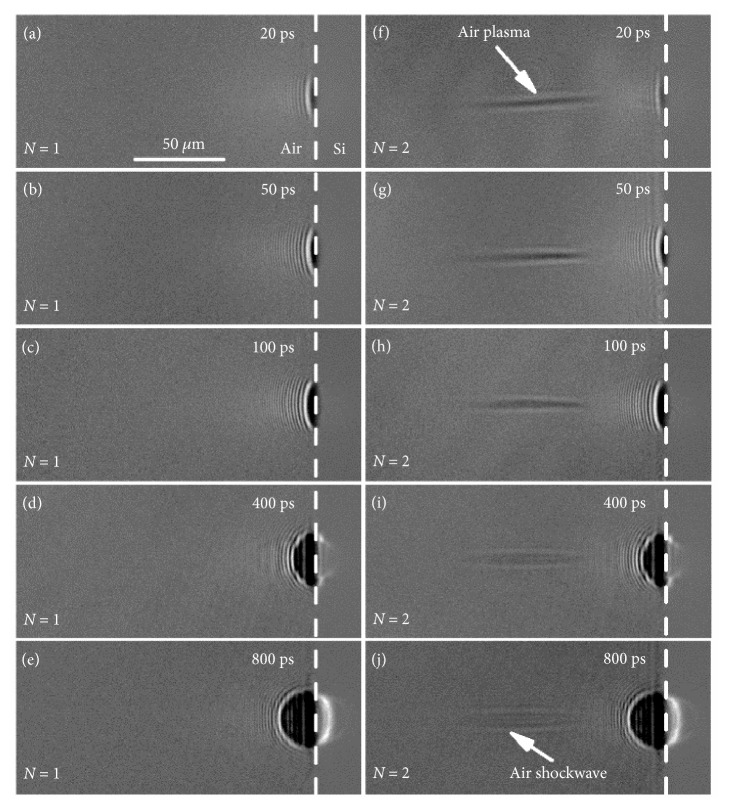
Time-resolved shadowgraphs of the plasma at probe delays on picosecond timescale. (a–e) Shadowgraphs for the first pulse. (f–j) Shadowgraphs for the second pulse. The laser fluence is 7.27 J/cm^2^. The dashed line indicates the interface of silicon and air.

**Figure 5:**
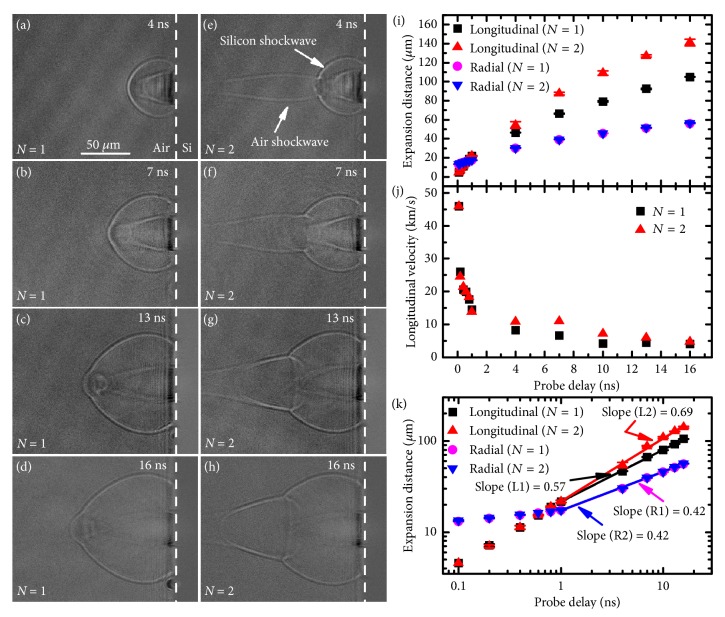
Time-resolved plasma shockwave expansion at probe delays on nanosecond timescale. (a–d) Shadowgraphs for the first pulse. (e–h) Shadowgraphs for the second pulse. The dashed line indicates the interface of silicon and air. (i) Measurements of longitudinal and radial expansion of the silicon shockwave as a function of time for the first two pulses ablation of silicon. (j) Calculated longitudinal velocities of silicon shockwave as a function of time for the first two pulses ablation of silicon. (k) Double logarithmic fitting of longitudinal and radial expansion for the first two pulses ablation. The laser fluence is 7.27 J/cm^2^.

The air plasma channel can affect the expansion of laser-induced plasma and the subsequent shockwave.  Figure 4 shows the transient shadowgraphs of the plasma induced by the first two pulses ablation of silicon at probe delays on picosecond timescale. Similar to the observation on femtosecond timescale, the air plasma channel was clearly observed on picosecond timescale during the second pulse irradiation (Figures 4(f)–4(j)). The air plasma channel remained constant at probe delay up to 100 ps (Figures 4(f)–4(h)). When the probe delay exceeded 400 ps, the air plasma decayed gradually and expanded radially; this induced a cylinder-like shockwave (air shockwave for short) above the silicon surface (Figures 4(i) and 4(j)). The plasma induced by laser ablation of silicon (silicon plasma) was observed at a probe delay of 20 ps (Figures 4(a) and 4(f)), which was two orders of magnitude later than the emergence time of air plasma. Comparisons of Figures 4(a)–4(e) and Figures 4(f)–4(j) revealed no distinct differences between the silicon plasma evolutions induced by the first two pulses at probe delays on picosecond timescale. Furthermore, the expansion distance of the silicon plasma was so small that the plasma front did not yet contact the air plasma channel, which enabled the plasma to retain its original expansion characteristics. With increasing probe delays, a shockwave was formed and the shockwave front contacted with the air plasma channel, which significantly affects the expansion of the shockwave.  Figure 5 shows the transient shadowgraphs of the shockwave induced by the first two pulses ablation of silicon at probe delays on nanosecond timescale; to be distinguished from the shockwave induced by air plasma (air shockwave), the shockwave induced by laser-induced silicon plasma expansion was represented as silicon shockwave for short. For the first pulse ablation, a small bulge existed on the shockwave front, which is attributed to the weak laser-induced air breakdown that cannot be detected on femtosecond-to-picosecond timescale with the applied technique. However, because of the generation of the evident air shockwave with a lower density in the channel [24–26], the front of the silicon shockwave induced by the second pulse was distorted in the longitudinal direction when it contacted the air plasma channel (Figures 5(e)–5(h)). Thus, the bulge on the front of the silicon shockwave was more distinct compared with that induced by the first pulse.

Figure 5(i) shows the expansion distance of the silicon shockwave as a function of probe delay for the first two pulses ablation; the longitudinal expansion velocity obtained by differentiating is presented in Figure 5(j). At 7 ns, the longitudinal expansion distances were 66.5 *μ*m and 87.5 *μ*m for the first and second pulse ablation, respectively; the corresponding velocities were 6.7 × 10^3^ m/s and 11.0 × 10^3^ m/s, respectively. The silicon shockwave was therefore enhanced in the longitudinal direction during the second pulse ablation, which is due to the acceleration effect of the laser-induced air plasma [24–26]. At larger probe delays (i.e., 16 ns), the longitudinal velocity difference for the first two pulses ablation declined. This may be attributed to the limitation of plasma length and the decay of the air plasma channel. In general, the time-dependent expansion of the laser-induced plasma and shockwave can be described by the Sedov-Taylor solution [20]:

(1) $$\begin{array}{c} R=\lambda {\left(\;\frac{E}{\rho }\;\right)}^{1/\left(\beta +2\right)}t^{2/\left(\beta +2\right)}, \end{array}$$

where* R* is the expansion distance,* λ *is a constant dependent on the specific heat capacity ratio and approximately equal to unity,* E* is the energy converted into the plasma state,* ρ* is the density of the undisturbed air,* t* is the expansion time (probe delay), and *β* describes the dimension of expansion (values of 1, 2, and 3 represent planar, cylindrical, and spherical propagation, respectively).  Figure 5(k) shows the double logarithmic fitting of longitudinal and radial expansion at probe delays larger than 1 ns for the first two pulses ablation. The slope in the radial direction remained constant at 0.42 (*β* ~ 3) for the first two pulses ablation, indicating that the radial expansion of the silicon shockwave was spherical and not influenced by the air plasma. However, the slope in the longitudinal direction increased from 0.57 (*β* = 1.51) to 0.69 (*β* ~ 1) for the first two pulses ablation, indicating that the longitudinal expansion of the silicon shockwave was altered to typically planar. These similarities and differences in expansion characteristics suggest that the laser-induced silicon plasma and shockwave are significantly affected by the excitation of air plasma during the second pulse ablation. And in the scenario, the expansion of laser-induced silicon plasma and shockwave during second pulse ablation is similar to that demonstrated by previous works for the single femtosecond laser ablation in the case of strong excitation of air plasma [24–26].

### 2.2. Multiple Pulses Ablation

**Figure 6:**
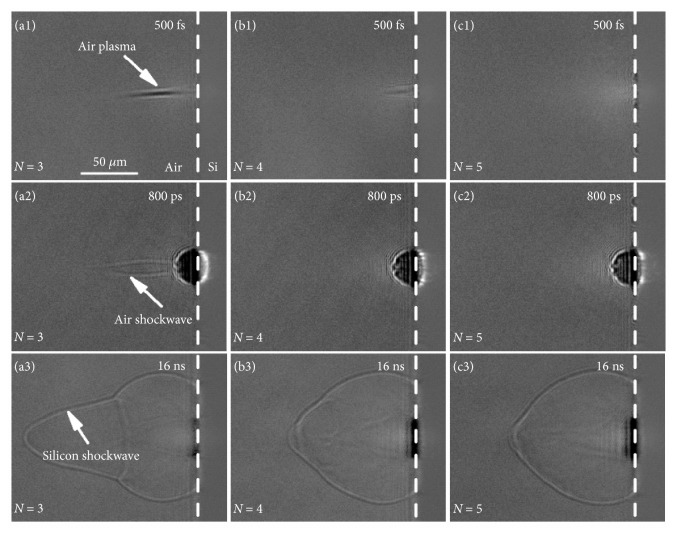
Shadowgraphs of the plasma shockwave at 500 fs, 800 ps, and 16 ns probe delays during multiple pulses ablation. (a1–a3) Shadowgraphs for the third pulse. (b1–b3) Shadowgraphs for the fourth pulse. (c1–c3) Shadowgraphs for the fifth pulse. The laser fluence is 7.27 J/cm^2^. The dashed line indicates the interface of silicon and air.

**Figure 7:**
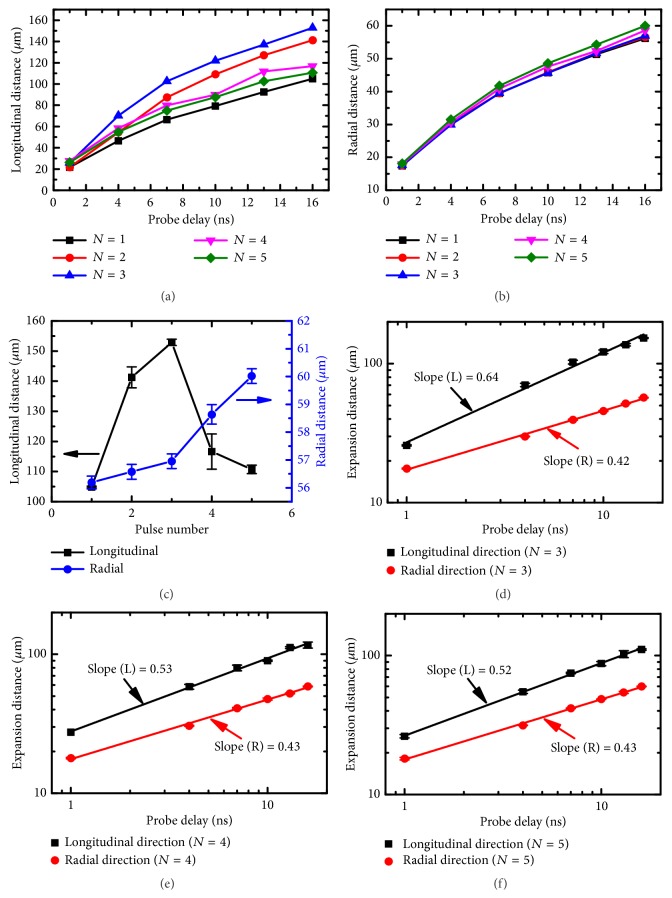
Pulse number-dependent expansion distance of the silicon shockwave. (a–b) Pulse number-dependent longitudinal and radial expansion of the silicon shockwave as a function of time. (c) Pulse number-dependent longitudinal and radial expansion of silicon shockwaves at a 16 ns probe delay. (d–f) Double logarithmic fitting of the longitudinal and radial expansion for the third, fourth, and fifth pulse ablation.

Previous studies have investigated the evolution of multiple pulses ablation and demonstrated the transition in the ablation surface from smooth to rough [31, 37, 38]. To study the effect of structure evolution on the plasma and shockwave dynamics, time-resolved shadowgraphs were recorded during multiple pulses ablation.  Figure 6 shows the typical shadowgraphs of the plasma and shockwave at probe delays on femtosecond, picosecond, and nanosecond timescales during the third, fourth, and fifth pulse ablation. During the third pulse ablation, air plasma was also observed at 500 fs probe delay (Figure 6(a1)). The length of the air plasma was shorter and positioned much closer to the sample surface compared with the air plasma induced by the second pulse, indicating that the crater induced by the second pulse had a smaller effective focal length. Afterwards, the air plasma made contact with the silicon plasma and enhanced the plasma and shockwave expansion (Figures 6(a2) and 6(a3)), which is similar to the observations during the second pulse ablation. However, when the fourth pulse was irradiated, the air plasma on femtosecond timescale faded quickly (Figure 6(b1)) and could barely be detected during the fifth pulse ablation (Figure 6(c1)). No evident air shockwave was observed on picosecond timescale, and the enhancement of the silicon shockwave in the longitudinal direction attenuated gradually. In particular, the morphology of the silicon shockwave induced by the fifth pulse (Figure 6(c3)) was similar to that induced by the first pulse (Figure 5(d)). The results indicate that the generation of air plasma is pulse number-dependent. Figures 7(a) and 7(b) depict the pulse number-dependent longitudinal and radial expansion of the silicon shockwave as a function of time. With the increase of pulse number, the expansion distance in the longitudinal direction increased rapidly to the maximum value when the third pulse is irradiated and then decreased gradually (Figures 7(a) and 7(c)). Although the expansion distance decreased rapidly, it was still larger than that induced by the first pulse. In addition, the expansion distance in the radial direction had a shaper increase after the fourth pulse irradiation (Figures 7(b) and 7(c)), indicating an increased energy deposition into the silicon plasma during multiple pulses ablation. These results signify another mechanism of shockwave enhancement in multiple pulses ablation. Furthermore, the expansion characteristics of multiple pulses ablation were investigated based on the Sedov-Taylor solution [20], as shown in Figures 7(d)–7(f). In the case of excitation of air plasma, the expansion of the silicon shockwave in the longitudinal direction was altered to a typically planar (one-dimensional) propagation and it was basically restored to its original expansion dimension as the air plasma vanished. Simultaneously, the expansion of the silicon shockwave in the radial direction remained nearly constant at a spherical (three-dimensional) propagation for the applied pulse number, showing no dependence on the excitation and vanishing of air plasma.

**Figure 8:**
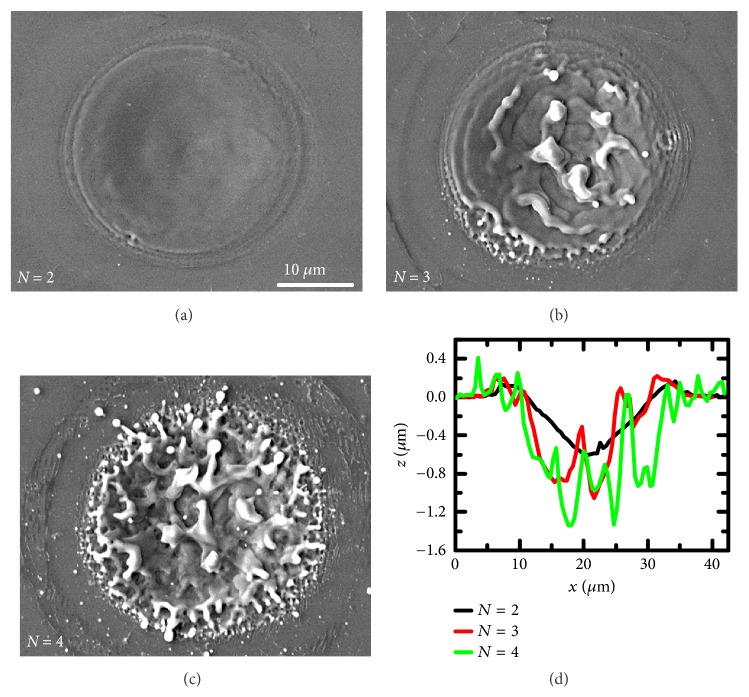
Silicon structure evolution during multiple pulses ablation. (a-c) SEM images of silicon structures induced by the second, third, and fourth pulses. (d) AFM profiles of the structure induced by the second, third, and fourth pulses. The laser fluence is 7.27 J/cm^2^.

To gain deeper insight into the pulse number-dependent plasma and shockwave evolution, the surface morphology was examined.  Figure 8 shows the silicon structures induced by the second, third, and fourth pulses; the structure induced by the first pulse is shown in Figures 3(a) and 3(b). Smooth crater-shaped structures were generated during the first two pulses ablation. Thus, these structures could reflect and refocus the next pulse, leading to the excitation of air plasma during the second and third pulse ablation and enhancement of the silicon shockwave expansion in the longitudinal direction. However, after the third pulse irradiation, the silicon surface became rough with some microbulges at the bottom of the crater (Figure 8(b)). This structure became rougher when the fourth pulse was irradiated; the periphery of the rough crater was covered with dense nanostructures. Craters with such micro/nanostructures fail to effectively focus the reflected laser during the next pulse irradiation, leading to the vanishing of air plasma during the fourth and fifth pulse ablation. In the scenario, the expansion of the silicon plasma and shockwave is dominated by the laser-material coupling. Silicon craters with micro/nanostructures can enhance laser-material coupling via antireflection effect and incubation effect. The antireflection effect increases the absorption of the incident laser with the light trapping effect, surface plasmon polaritons excitation, and effective medium effect [39–41]; the incubation effect due to surface defects is beneficial to material ablation [42–44]. The enhanced laser-material coupling can increase the energy deposition in the silicon plasma and thus result in stronger plasma and shockwave expansion observed before. Moreover, the enhancement by increased laser-material coupling is more isotropic in comparison with the anisotropic expansion characteristic enhanced by air plasma.

## 3. Discussion

In summary, we have investigated the laser-induced plasma dynamics during multiple femtosecond laser pulses ablation of silicon, which was significant but always ignored in previous studies. The structure-mediated excitation of air plasma was directly observed during multiple femtosecond laser pulses ablation. Furthermore, the expansion of plasma and shockwave was systematically analyzed, revealing two fundamental mechanisms for the plasma and shockwave expansion: the excitation of air plasma and laser-material coupling. These two mechanisms were found to be strongly dependent on the laser-induced surface structure. At small pulse number, smooth crater was generated by prior pulses, which can reflect and refocus the next pulse, inducing higher laser intensity above the sample surface. Thus, air plasma was excited and dominated the anisotropic expansion of the silicon plasma and shockwave. At higher pulse number, the smooth crater became rough covered with micro/nanostructures, which was unable to refocus the incident pulse. In the scenario, the air plasma vanished and the laser-material coupling was instead the core mechanism for the expansion of the silicon plasma and shockwave with more isotropic characteristics. Our findings indicate the underlying mechanisms of laser-induced plasma dynamics during multiple pulses ablation, and they are of fundamental importance for deep insight into the nature of ultrafast laser-material interactions.

## 4. Materials and Methods

### 4.1. Femtosecond Pump-Probe Shadowgraphy

The ultrafast pump-probe shadowgraphy was performed with a commercial Ti: sapphire femtosecond laser regenerative amplifier (Spitfire, Spectral Physics), which delivers 50 fs pulses with a central wavelength of 800 nm. Figure 1 shows the schematic of the pump-probe setup. During the experiment, the laser system was operated in external gated mode and a time controller was employed to trigger a single pulse. The emitted pulse was split into pump and probe pulses. The pump pulse was focused normally onto the silicon surface (optical polishing, orientation (100)) using a plano-convex lens (*f* = 100 mm), generating a Gaussian intensity distribution with a diameter of approximately 35 *μ*m (1/e^2^-decay distance from peak value). The laser fluence (peak fluence) herein was set at a constant of 7.27 J/cm^2^, which was much larger than the ablation threshold of silicon. The probe pulse, after passing an optical delay line (time uncertainty < 20 fs), was frequency doubled with a BBO to 400 nm, and a dielectric coating short-pass filter (edge wavelength = 650 nm, Daheng Optics) was used to block the residual fundamental 800 nm pulse. The probe pulse was then directed to illuminate the interaction region along the direction perpendicular to the pump pulse and parallel to the sample surface. The transmitted probe pulse was collected with an objective (20×, NA = 0.45, Olympus) and directed onto a charge-coupled device (CCD). A 400 nm dielectric coating band-pass filter (full width half maximum = 10 nm, Thorlabs) was added before the CCD to suppress the background illumination. Before silicon ablation experiment, the zero-delay was adjusted by probing laser-induced air plasma at high laser fluence with the sample being removed, which was set as the time the air plasma was detected at the focal spot. To study the ablation dynamics of multiple pulses irradiation, a series of shadowgraphs of plasma and shockwave induced by successive pulses were recorded at probe delays ranging from femtosecond to nanosecond timescale. During multiple pulses irradiation, the pump pulse separation exceeded one second. To improve the contrast of the obtained shadowgraphs, image background subtraction was performed by subtracting the background image from the obtained pump-probe images [45]; the background image was recorded with the pump pulse being blocked. The appearance of the silicon (right area of the dashed line in the shadowgraphs) after background subtraction looks different from the original black appearance.

### 4.2. Characterization of Surface Morphology

The ablation morphology was characterized with a scanning electron microscope (XL30 S-FEG, FEI) and an atomic force microscope (Dimension edge, Bruker). The light focusing ability of the silicon-based crater generated by the first pulse was characterized with an optical system shown in Figure 3(c). The reflected light of the crater was collected with a lens and imaged on a CCD.

## Data Availability

All data needed to evaluate the conclusions in the paper are present in the paper. Additional data related to this paper may be requested from the authors.
